# Comprehensive School Physical Activity Programming and Health Behavior Knowledge

**DOI:** 10.3389/fpubh.2020.00321

**Published:** 2020-07-24

**Authors:** Rose E. Mattson, Ryan D. Burns, Timothy A. Brusseau, Julie M. Metos, Kristine C. Jordan

**Affiliations:** ^1^Department of Nutrition and Integrative Physiology, University of Utah, Salt Lake City, UT, United States; ^2^Department of Health and Kinesiology, University of Utah, Salt Lake City, UT, United States

**Keywords:** child, community, exercise, health, physical activity, school

## Abstract

No study to date has examined the effect of a multicomponent school-based physical activity program on health behavior knowledge in a large sample of low-income children from the US. The purpose of this study was to explore the change in physical activity and nutrition knowledge during a Comprehensive School Physical Activity Program (CSPAP) in children. Participants were a convenience sample of 789 children recruited from the 4th to 6th grades from five low-income Title I schools located within the Mountain West Region of the US. Students completed two questionnaires consisting of a physical activity and a nutrition knowledge assessment. Questionnaires were administered at baseline before the commencement of CSPAP and at a 36-week follow-up. Data were analyzed using a 3 × 2 × 2 doubly MANOVA test. Physical activity knowledge scores significantly improved from pretest to posttest during the intervention (*p* = 0.045, Cohen's *d* = 0.18). Grade level modified the time effects, with older children in grades 5 and 6 displaying greater improvements in physical activity knowledge than younger children in grade 4 (*p* = 0.044, Cohen's *d* = 0.33). There were no significant improvements in nutrition knowledge scores during the CSPAP (*p* = 0.150). These findings demonstrate that improvements in physical activity knowledge can occur during a multicomponent school-based intervention. Improvements in physical activity knowledge may translate to improvements in habitual physical activity behaviors and positively influence children's health outcomes, especially in older children.

## Introduction

Childhood obesity is a significant public health issue in the United States. According to the US Centers for Disease Control and Prevention (CDC), 17% of children and adolescents ages 2–19 are obese (1). Obesity rates in children ages 6–11 have increased from 7% in 1980 to 18% in 2012 (1) Similarly, adolescent obesity rates for ages 12–19 have increased from 5% in 1980 to 21% in 2012 (1). In children and adolescents, obesity is associated with an increased risk of high cholesterol, high blood pressure, pre-diabetes, bone and joint problems, physiological and social problems, and sleep apnea (2). Childhood overweight and obesity often predicts adult obesity and its associated morbidity (3). Regarding educational outcomes, Carey et al. (4) found that obese children were significantly more likely to have school absences, school-related problems, and lower involvement in school activities, as compared to normal weight children.

The prevalence of childhood obesity is higher in minority and low socioeconomic (SES) populations. In 2009–2010, Ogden et al. (5) reported obesity prevalence rates in children and adolescents by ethnicity as follows: Hispanic (21.2%), non-Hispanic black (24.3%), and non-Hispanic white (14.0%). Low SES minority groups often have the highest health needs (3, 6). Between 1998 and 2010, obesity in low SES children significantly increased while rates declined in children from higher SES backgrounds (7). Specifically, children below the poverty line have 83% higher odds of obesity (7, 8). Additionally, it has been reported that obesity prevalence is higher in children from households with lower levels of education and non-English speaking families (8–10).

Schools provide a unique opportunity for obesity prevention programs due to the amount of time children spend in schools and the ability to impact a large population of children (3). First, children spend nearly 900 h of instruction time annually in schools (11). Second, in the Fall of 2016, there were ~50.4 million students enrolled in primary and secondary schools, with an estimated 35.4 million students in prekindergarten to grade 8 (12). Furthermore, children who learn positive health behaviors at an early age may have decreased rates of obesity and overweight later in life (13).

School-based physical activity education interventions have reported improvements in Body Mass Index (BMI), cardio-respiratory endurance, and physical activity (14–16). Li et al. (15) detailed a 12-week physical activity program with physical education motor skill development, activities for overweight/obese children, the inclusion of physical activity at home, and health education lectures. Results showed that children in the intervention group had a significant decrease in BMI and a significant increase in moderate-to-vigorous physical activity (MVPA) (16). Similarly, increases in health-related fitness knowledge may translate to increased physical activity levels of high school students (16). Regarding barriers, Longmuir et al. (17) reported that children ages 8–12 with reduced physical literacy may be less likely to participate in physical activity. Additionally, children from low socioeconomic families have added barriers that may prevent them from participating in physical activity (18, 19).

School-based nutrition education interventions have demonstrated significant improvements in nutrition knowledge. Carraway-Stage et al. (20) integrated science and nutrition in the 4th grade curriculum. Results indicated that intervention students displayed a significant increase in nutrition knowledge, as compared to controls. Turnin et al. (21) utilized an interactive software program as a nutrition intervention for middle school children. After one school year, study findings included a decline in BMI z-scores and increased consumption of fruits, vegetables, dairy, and starchy foods. Habib-Mourad et al. (22) delivered classroom educational sessions for 9–11-year olds, with the focus on increasing fruit and vegetable consumption, increasing MVPA, and identifying healthy snacks and drinks. At post-intervention, nutrition knowledge scores, and self-efficacy scores increased significantly from baseline after 12 weeks. Other programs that included a nutrition education intervention demonstrated improvements in nutrition knowledge and also showed reductions in body weight (23).

Comprehensive nutrition and physical activity interventions have been associated with reductions in childhood overweight and obesity (24, 25). Maatoug et al. (26) provided education sessions on the importance of fruit and vegetable consumption, benefits of physical education, and how to increase physical activity into daily life in a 3-year school-based intervention. Results indicated a decline in the percentage of obese children (7–6.5%) in the intervention group, as compared to the increase in the percentage of obese children (4.5–6.9%) in controls. Also, children in the intervention group ate more fruits and vegetables, as compared to controls (26). Nemet et al. (25) found that in a physical activity, nutrition education, and behavioral health intervention, obese children had a decreased BMI, body fat percentage, and cholesterol levels after a 3-month intervention, in comparison to controls. In a 1-year follow up, intervention children continued to have significant differences in body weight, BMI, and body fat percentage in comparison to the control group (25). Another elementary school intervention utilized a Comprehensive School Physical Activity Program (CSPAP), with observed improvements in the food environment, as well as educational strategies in an intervention for kindergarten through 5th grade children (27). Study findings showed a 15.2% decrease in childhood obesity over a 6-year span (27). Despite these findings, no study to date has explored the effect of CSPAP on physical activity and nutrition knowledge in a sample of children from low-income schools. Therefore, the purpose of this study was to analyze change in both physical activity and nutrition knowledge scores during a CSPAP intervention. We hypothesized that there would be a statistically significant difference between pre- and post-physical activity knowledge and nutrition knowledge.

## Methods

### Participants

The participants were a convenience sample of children (*N* = 789) from five low-income elementary schools from the Mountain West Region of the US. Children from the 4th−6th grades were asked to participate. Using information from the school district website, 91% of children were of an ethnic minority and 60% of children were from low-income families (28). Of the participants, girls and boys were enrolled in 4th (47%), 5th (41%), and 6th (12%) grade. Demographic data are presented in Table 1. Prior to data collection, written consent was acquired from the students and written assent was obtained from the parents. The University Institutional Review Board approved all protocols used in this study.

**Table 1:** Demographic data showing distribution of participants across sexes and grade levels.

	**Grade 4**	**Grade 5**	**Grade 6**	**Total**
**Female**	219	165	50	434
**Male**	152	159	44	355
**Total**	371	324	94	789

### Protocol

The CSPAP schools hired Physical Activity Leaders (PAL) that had the main responsibility of working with school personnel to improve physical activity infrastructure and encourage activity throughout the day. PALs were trained by the same school district personnel within the Healthy Lifestyles Department and were provided the same professional development opportunities (e.g., development seminars) throughout the duration of the intervention. The CSPAP's primary focus was to provide training and assistance to improve the quality of physical education, recess, and classroom-based physical activity opportunities at each of the three schools. Specifically, monthly in-service opportunities for classroom teachers, physical educators, and physical activity supervisors were provided to ensure that physical activity opportunities in physical education, at recess, and the classroom were student-centered and developmentally appropriate. A goal set by the school district was for teachers and school personnel to maximize physical activity opportunities through greater student engagement, improved lesson planning, and decreased management and waiting time. In addition to improving the quality of physical education, CSPAP offered physical activity opportunities throughout the school day during specific leisure times and integrated physical activity into lessons and classroom activity breaks.

The physical activity and nutrition education intervention occurred during the 2015-2016 school year. PALs worked with classroom and PE teachers to best implement the CSPAP. Physical activity and nutrition knowledge were integrated into PE lessons and classroom activity breaks through activities and lessons. Additionally, PALs created bulletin boards, handouts, and other educational materials for the intervention. Consistency of these promotion methods across schools were tracked via biannual reports from each PAL provided to the school district personnel and research team. No other health behavior intervention was currently being implemented at any of the schools, confirmed by the school district's Healthy Lifestyles Department.

The physical activity knowledge survey was tested utilizing the PE Metrics physical education activity test which was developed by the Society of Health and Physical Educators (SHAPE) (29). This test provides assessments for the National Standards for Physical Education including the following: motor skills and movement patterns, comprehension of movement concepts and strategies, qualities of an active lifestyle, and student knowledge of appropriate behavior during physical activity (29). The PE Metrics test consists of 28 questions organized into specific performance descriptors relating to health-related fitness knowledge (30). Hodges et al. (30) found PE Metrics to be a valid and reliable tool in evaluating health-related fitness knowledge. Students took the test online during school hours once in October and again in May. The scores were analyzed as the proportion of questions answered correctly. For the nutrition knowledge survey, students completed a computer-based multiple-choice assessment in ~10–15 min. The 15-item survey focused on the following domains: types of healthful foods, food functions, and food groups. Scores were calculated by adding the total of correct answers and then dividing by 15 for percentage correct (30).

### Statistical Analysis

Data were screened for outliers using box plots and z-scores. Histograms and Q-Q plots were used to test for Gaussian distributions of the physical activity and nutrition knowledge variables. The distributions of the knowledge outcome variables were approximately Gaussian, characterized by low variability (see Table 2). Because proportion data is bounded by [0,1], statistical assumptions may be violated, thus warranting data transformation (31). However, given the approximately Gaussian distributions of the knowledge outcome variables, low score variability, and mean proportion scores ranging from 0.2 to 0.8, it was decided to analyze untransformed data within the general linear model (31). This also better facilitates interpretation of scores compared to the use of transformed data (31). Descriptive statistics [means (M) and standard deviations (SD)] were reported for each dependent variable. To test the hypothesis that physical activity knowledge and nutrition knowledge scores would increase during the CSPAP intervention, and to examine the modifying effects of grade level and sex, a three-factor 3 × 2 × 2 mixed-design doubly Multivariate Analysis of Variance (MANOVA) test was employed. If a statistically significant multivariate model was found, follow-up univariate Analysis of Variance (ANOVA) tests were employed. For each dependent variable, statistically significant time main effects were reported as well as any statistically significant 2-way or 3-way interactions. The dependent variables were physical activity knowledge scores and nutrition knowledge scores. The independent factors were grade level (4th−6th), sex (girls, boys), and time (pretest, posttest). Levene's Test was used to test the assumption of homogeneity of variance. Bonferroni *post hoc* tests were utilized if there were any statistically significant differences among grade levels. Effect sizes were calculated using Cohen's delta (d), with *d* < 0.20 indicating a small effect size, *d* = 0.50 indicating a medium effect size, and *d* > 0.80 indicating a large effect size. An a priori alpha level was set at *p* < 0.05 and the data were analyzed using SPSS v25.0 (Armonk, New York, USA).

**Table 2:** Pre- and post-intervention comparisons between physical activity and nutrition knowledge scores.

	**Pretest (mean ± SD)**	**Posttest (mean ± SD)**	**Difference score (mean ± SD)**	***P*-value**	**Effect size**
**Physical activity knowledge**	0.26 ± 0.11	0.28 ± 0.12	0.02 ± 0.01^*^	0.045	0.18
**Nutrition knowledge**	0.73 ± 0.12	0.74 ± 0.14	0.01 ± 0.002	0.150	0.08

*Scores reflect proportion of answers correct*;

* *p < 0.05 for significant differences between pretest and posttest scores*.

## Results

**Figure 1:**
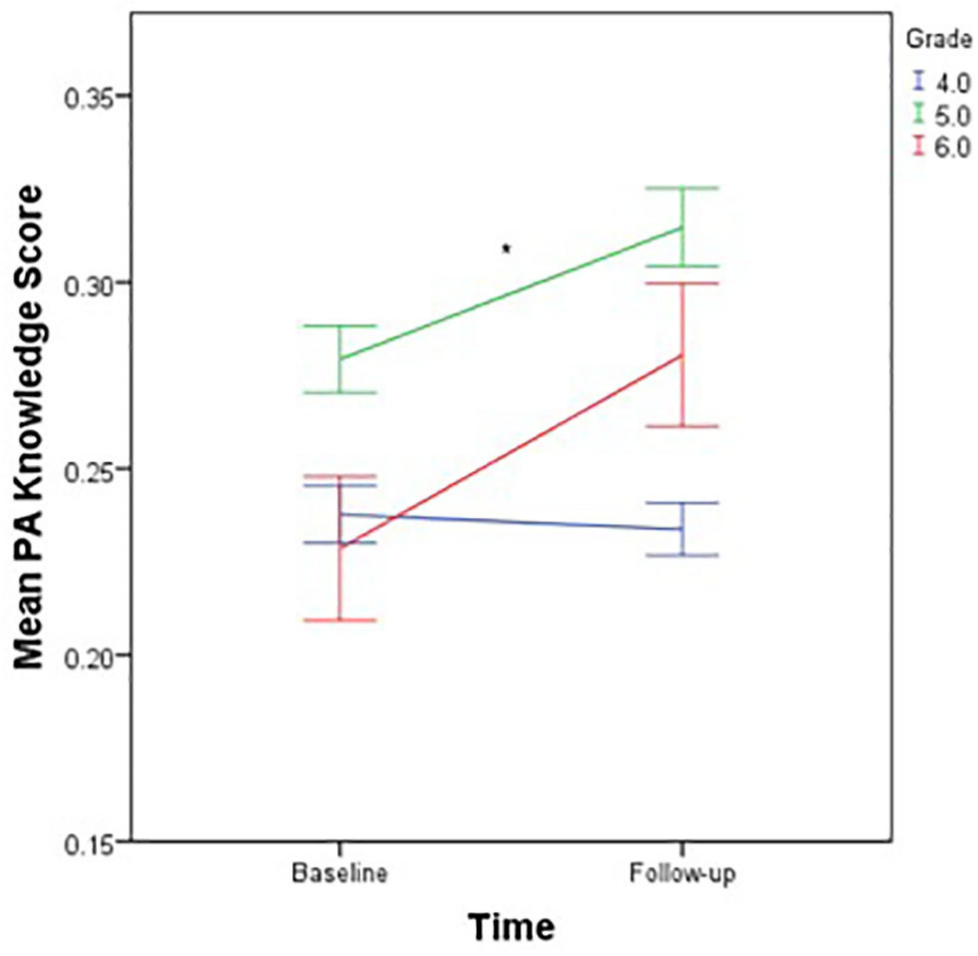
Mean physical activity knowledge scores by time-point, stratified by grade level. Mean PA Knowledge Score, Mean physical activity knowledge score; scores are communicated as a proportion of answers correct; *p < 0.05 for significant differences in physical activity knowledge change scores among grade levels.

Pretest and posttest statistics are presented in Table 2. All 789 children submitted completed questionnaires at follow-up. There was no missing data. The MANOVA model was statistically significant (Wilks' λ = 0.49, *p* = 0.048). With physical activity knowledge as the dependent variable, and time, sex and grade level as independent variables, a follow-up ANOVA test revealed that physical activity knowledge scores increased significantly from pre- intervention to post-intervention (*p* = 0.045; see Table 2 and Supplementary Figure 1), however this difference represented a small-sized effect (*d* = 0.18). The sex main effect was statistically significant (*F* = 4.10, *p* = 0.040) with female students achieving higher scores than males. Supplementary Figure 2 visually displays the sex differences in nutrition knowledge scores pooled across time-points. The grade level main effect was also statistically significant (*F* = 17.93, *p* < 0.001); statistical differences were noted between 5th and 6th graders and between 5th graders and 4th graders (*p* < 0.05) with 5th graders scoring higher on the physical activity knowledge scores (Supplementary Figure 2). Interestingly, there was also a statistically significant grade × time interaction on physical activity knowledge scores (*F* = 3.27, *p* = 0.044). The average 4th grade physical activity knowledge scores were primarily stable from pretest to posttest (*M* = 0.24) while both 5th and 6th graders mean scores increased from pretest to posttest (*M* = 0.28–0.32 and *M* = 0.24–0.28, respectively; see Figure 1). The differences in the change scores among grade levels represented a small-to-medium effect size (*d* = 0.33).

With nutrition knowledge as the dependent variable, and time, sex, and grade level as independent variables, a follow-up ANOVA test found that nutrition knowledge scores slightly increased from *M* = 10.96 to *M* = 11.17, however this change was not statistically significant (*p* = 0.150). Both grade level (*F* = 5.68, *p* < 0.001) and sex (*F* = 5.33, *p* = 0.022) main effects were found to be statistically significant with girls scoring higher than boys (see Supplementary Figure 3). Additionally, 5th grade students had significantly higher nutrition knowledge scores than 4th grade students when pooled across both time-points (*p* = 0.005; Supplementary Figure 4).

## Discussion

The purpose of this study was to examine the effect of a CSPAP on health behavior knowledge in a sample of low-income school-aged children. This study was part of a larger intervention also examining school-based physical activity behaviors, health-related fitness, and gross motor skills (32). Physical activity knowledge scores significantly increased from pre- to post-intervention. The current study results support previous findings of improvements in physical activity knowledge during school-based programs (33, 34). Moreover, our results are comparable to Tsia et al. (35), as that study reported increases in physical activity knowledge among ethnic minority low SES children in a school-based intervention (35). Overall, the literature supports the importance of incorporating physical activity education in a comprehensive school-based intervention. Table 3 summarizes pertinent findings on improving health behavior knowledge though the use of school-based physical activity and nutrition programming.

**Table 3:** Comparison of study results to other school-based interventions for improving health behavior knowledge.

**Study**	**Intervention**	**Knowledge variables**	**Findings**	**Effect sizes**
Kaufman-Shriqui et al. (36)	Israel school-based intervention	Nutrition knowledge	Higher nutrition knowledge	Large
Lakshman et al. (37)	TOP GRUB card game	Nutrition knowledge	Higher nutrition knowledge	Medium
Mattson et al. [^†^]	CSPAP	Physical activity and nutrition knowledge	Higher physical activity knowledge	Small
Mihrshahi et al. (33)	Good start	Physical activity and nutrition knowledge	Higher physical activity and nutrition knowledge	Large
Saksvig et al. (32)	Sandy Lake school diabetes prevention	Nutrition knowledge	Higher nutrition knowledge	Large
Tsia et al. (34)	TAKE10!	Physical activity and nutrition knowledge	Higher physical activity and nutrition knowledge	Medium

† *Denotes current study*.

The physical activity knowledge results also yielded significant differences between the 5th and 6th grades, as well as between the 4th and 5th grades. Students in the 5th grade scored higher than both 4th and 6th grade students. These results could be explained by Hodges et al. (30) who found the PE Metrics test to be a valid tool for 5th grade students, with consideration that the tool may not be applicable to all age groups. Additionally, 4th grade scores decreased from pre- to post-intervention, while both 5th and 6th grade scores increased from pre- to post-intervention. In a review, Budd and Volpe (38) reported that children of older age often respond better to interventions, as compared to younger children. Specific to physical activity and nutrition knowledge, younger children may have difficulty conceptualizing topics pertaining to health behaviors and health outcomes. There may even be discordance in overall interest in these topics when comparing younger and older children. This phenomenon has also been seen in other concepts such as physical activity goal setting (39). Therefore, even though only a few years separate 4th and 6th graders, school-based programming aiming to improve health behavior knowledge may need to be tailored to specific grade levels to account for possible discordance in health behavior conceptualization and interest. However, higher levels of physical activity knowledge gained in older children relative to younger children is still a positive finding given that physical activity levels tend to drastically decline throughout the school year in middle school-aged students (40).

Additionally, there was a difference in knowledge scores by sex, with females scoring higher than males. This finding may be explained by a review of school-based obesity prevention programs (41). Thus, study outcomes based on sex may be accounted for by the type of physical activity knowledge intervention. Despite this, there was no statistically significant sex by time interaction observed in the analysis of the current study, suggesting that the relationship between CSPAP and health behavior knowledge is not influenced (i.e., moderated) by sex. Also, higher physical activity knowledge may not transfer to higher levels of physical activity behavior, as the literature has consistently shown boys tending to yield higher levels of physical activity during elementary and middle school than girls, even though these differences tend to be only modest in magnitude (42, 43).

The CSPAP intervention demonstrated increases in nutrition knowledge scores; however, contrary to our hypothesis, this increase was not statistically significant. Current research demonstrates that nutrition education interventions result in improvements in knowledge, thus, our results are inconsistent with previous research. Similarly, Gower et al. (44) reported that 1st−4th grade students significantly improved nutrition knowledge scores upon completion of a 4-week intervention, as compared to controls. While the computerized testing method was similar to the current study, Gower et al. (44) implemented detailed lesson plans, with 4 weekly nutrition education classes. Another nutrition program for low SES minority children showed significant improvements in nutrition knowledge after a substantial intervention focused on classroom lesson plans, posters, food pictures, student workbooks, and parental homework (45). A further intervention utilized a game-based educational format to yield significant increases in nutrition knowledge (46). Therefore, the current results could be explained by the limited nutrition education implemented.

Additionally, there were significant differences in nutrition knowledge scores between sexes, with females scoring higher than males. These results are in contrast to previous findings in the literature. Warren et al. (47) found no differences between the sexes in a 14-month school-based nutrition education intervention for children ages 5–7 (*N* = 213), with overall nutrition knowledge improvements of 15 percent. However, the sex differences observed in the current study were pooled over both measurement time-points (i.e., a sex main effect) and, like physical activity knowledge, there was no sex by time interaction observed in the analysis. This suggests that although females may have had overall higher nutrition knowledge, sex did not influence the relationship between CSPAP and change in nutrition knowledge scores from pretest to posttest. Furthermore, sex differences were relatively small in magnitude, with effect sizes being characterized as small (*d* = 0.14). Despite these small differences, girls may have a better conceptualization of the relationships between health behaviors and health outcomes compared to boys. Like grade level differences, girls may also have greater interest in these topics compared to boys within a respective age range or grade level. This may influence effectiveness of programs within school settings. Indeed, school-based programs aimed to reduce cardiometabolic risk has shown greater effectiveness in girls compared to boys (47).

Despite the positive findings related to physical activity knowledge, the improvements were small in magnitude across the duration of the intervention. Compared to other interventions, the knowledge improvements observed in the current study are not as drastic (see Table 3). The goal of CSPAP is to provide enhanced and extended opportunities for physical activity behaviors; however, it is unknown whether small improvements in knowledge gained from CSPAP transfer to meaningful improvements in movement behaviors in children. It is also unknown whether any gains in knowledge track after conclusion of a health behavior intervention. Most school-based multicomponent interventions have yielded small improvements in actual physical activity behavior when data are analyzed at the group level (36). Therefore, is logical that knowledge gained from multicomponent interventions would also be small in magnitude. However, individual-level analyses may yield different results as children with very low physical activity behavior and/or knowledge at baseline may greatly benefit from multicomponent programs such as CSPAP. Examining additional effect modifiers is a priority for future research.

There are several limitations to this study, including the following: the use of a convenience sample, the physical activity and nutrition intervention, the knowledge assessments, and the potential confounding variables. First, this study utilized a convenience sample, which may be prone to bias and under- or over-representation of groups within the sample. Second, the physical activity and nutrition knowledge intervention was minimal, consisting of education implemented into PE lessons, classroom activity breaks, and informative bulletin boards. Third, culturally appropriate foods were not used in the nutrition knowledge survey, which may have led to confusion among study participants. Additionally, the knowledge assessments were the same for all grade levels and for non-English speakers, which may have contributed to skewed results. Fourth, direct physiological fitness assessment was not collected concurrently with the knowledge data. This should be a priority for future research. Finally, potential confounding variables, such as previous physical activity level, history of disease, and dietary habits, were not included in the data analysis.

This study provides evidence that school-based interventions in an ethnic minority low socioeconomic population should include three particular directives. First, our study demonstrated that school-based interventions should include a physical activity education component. Physical activity knowledge may result in reductions in BMI and improvements in aerobic fitness. Second, these interventions would benefit from a nutrition education section. Nutrition education interventions may be more effective if utilizing multiple modalities such as classroom lesson plans, interactive games, and student homework. We recommend a multicomponent school-based intervention to help decrease rates of childhood obesity in ethnic minority low SES populations.

## Data Availability Statement

The datasets generated for this study are available on request to the corresponding author.

## Ethics Statement

The studies involving human participants were reviewed and approved by University of Utah Institutional Review Board. Written informed consent to participate in this study was provided by the participants' legal guardian/next of kin.

## Author Contributions

RM, TB, JM, and KJ conceived the study, collected data, wrote the initial draft of the manuscript, and approved the manuscript for submission. RB performed the data analysis, wrote the initial draft of the manuscript, and approved the manuscript for submission. All authors contributed to the article and approved the submitted version.

## Conflict of Interest

The authors declare that the research was conducted in the absence of any commercial or financial relationships that could be construed as a potential conflict of interest.
