# Supplementary material for: Comprehensive School Physical Activity Programming and Health Behavior Knowledge
Source: Front Public Health. 2020 Jul 24;8:321. doi: 10.3389/fpubh.2020.00321 (PMC7393515; doi:10.3389/fpubh.2020.00321)
Supplement: Supplementary file 1 [file Data_Sheet_1.docx]

**
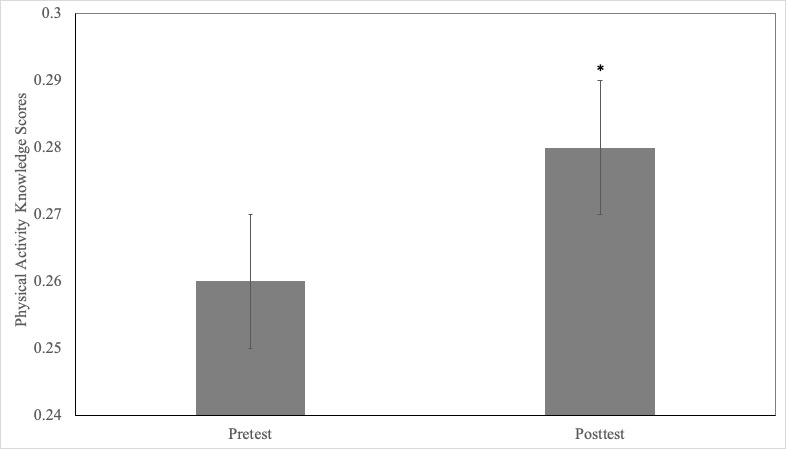
**

**Supplementary Figure 1.** Mean physical activity knowledge scores by time-point.

*Note:* Scores are communicated as a proportion of answers correct; * *p* < 0.05 for significant differences in physical activity knowledge scores between time-points.

**
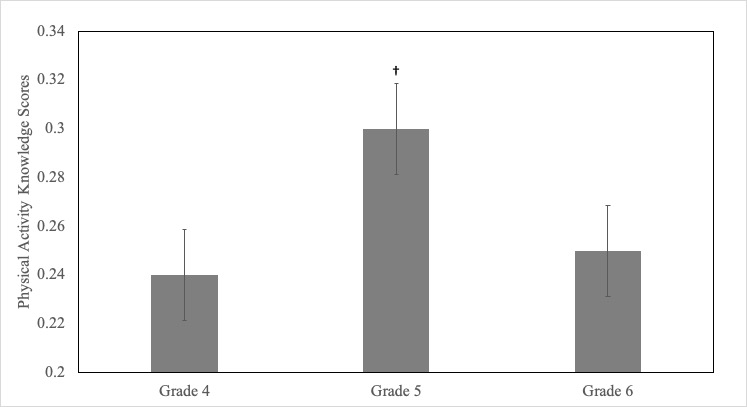
**

**Supplementary Figure 2.** Mean physical activity knowledge scores among grade levels.

*Note:* Scores are communicated as a proportion of answers correct; scores pooled across both time-points; † *p* < 0.05 for significant differences in physical activity knowledge scores compared to grade 4 and grade 6.

**
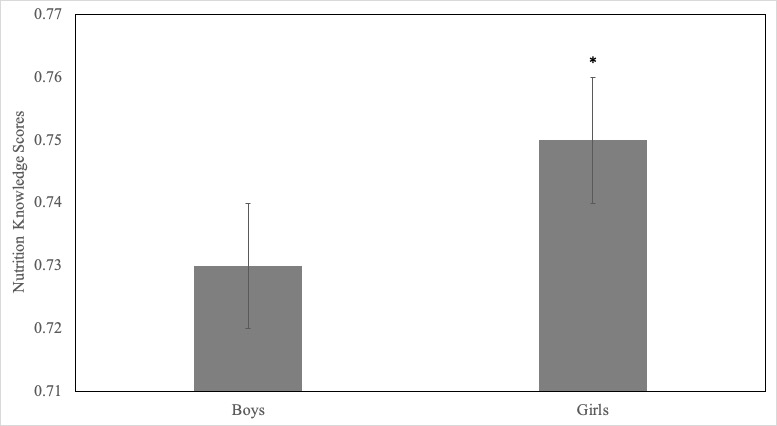
**

**Supplementary Figure 3.** Mean nutrition knowledge scores by sex.

*Note:* Scores are communicated as a proportion of answers correct; scores pooled over both time-points; * *p* < 0.05 for significant differences in nutrition knowledge scores between sexes.

**
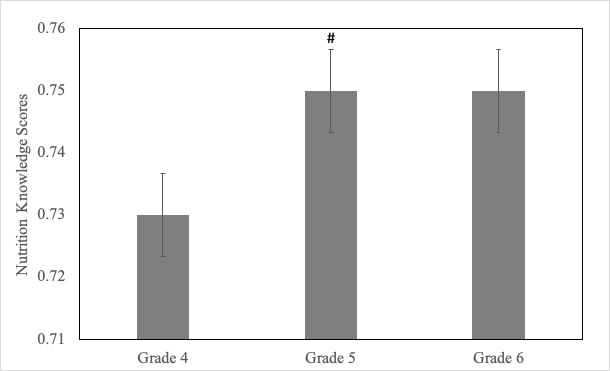
**

**Supplementary Figure 4.** Mean nutrition knowledge scores among grade levels.

*Note:* Scores are communicated as a proportion of answers correct; scores pooled over both time-points; # *p* < 0.05 for significant differences in nutrition knowledge scores compared to grade 4.
